# 1802. Reconstructing Twelve Years of Dengue Outbreaks in the Caribbean Using Travel Surveillance

**DOI:** 10.1093/ofid/ofad500.1631

**Published:** 2023-11-27

**Authors:** Emma Taylor-Salmon, Andrea Morrison, Nathan D Grubaugh

**Affiliations:** Yale School of Medicine, New Haven, Connecticut; Florida Department of Health, Tallahassee, Florida; Yale School of Public Health, New Haven, Connecticut

## Abstract

**Background:**

Dengue is the most common arboviral disease worldwide and the leading cause of febrile illness among US travelers returning from the Caribbean. Infectious disease surveillance of international travelers is an effective method for detecting pathogens circulating in resource-limited areas. Due to the large number of infected travelers diagnosed in Florida, we hypothesized that we could use travel surveillance data to reconstruct local outbreaks in the Caribbean, focusing on Cuba, Dominican Republic, Haiti, Jamaica, and Puerto Rico.

**Methods:**

We obtained reports of international travel-associated dengue virus infections diagnosed in Florida between 2009-2022 from the Florida Department of Health (FL DOH) mosquito-borne disease surveillance system. We calculated travel incidence rates using the yearly number of air travel passengers arriving in Florida from international destinations. We calculated local incidence rates from yearly dengue case counts from Caribbean countries and territories with local transmission, provided by the Pan-American Health Organization (PAHO).

**Results:**

Most infected travelers recently arrived from Cuba; however, travel dengue incidence poorly correlated with local dengue incidence there (R = 0.502), raising concerns about the accuracy of local surveillance. Conversely, travel dengue incidence from the Dominican Republic, Jamaica and Puerto Rico strongly correlated with local incidence (R = 0.744, 0.942, and 0.930), implying that travel data is a good predictor of local outbreaks in these countries. We discovered that during outbreak years different dengue virus (DENV) serotypes were responsible for outbreaks on each island, demonstrating that regional outbreaks are likely due to overall favorable conditions, such as mosquito abundance, and not due to a new introduction or mutation.

**Conclusion:**

Travel surveillance data provides a good approximation of local dengue incidence in some Caribbean countries but does not correlate strongly with local case reporting from Cuba and Haiti. This raises the possibility of using real-time tracking and sequencing of dengue cases in Florida, paired with travel surveillance data, to detect Caribbean outbreaks as they are occurring to inform public health policy in resource-limited countries.

**Disclosures:**

**All Authors**: No reported disclosures

